# Endometriosis: the ongoing quest for therapeutic modulators to prevent cardiovascular adverse outcomes

**DOI:** 10.1093/ehjopen/oeaf044

**Published:** 2025-04-18

**Authors:** Benjamin Marchandot, Olivier Morel

**Affiliations:** Department of Cardiology, University Hospital of Strasbourg, Nouvel Hôpital Civil, 1 place de l’Hôpital, BP 426-67091, Strasbourg 67000, France; Research Unit - UR3074, Translational Cardiovascular Medicine, University of Strasbourg, Strasbourg, France; Groupe pour l’Enseignement et la Recherche Cardiovasculaire en Alsace (GERCA), Strasbourg, France; Department of Cardiology, University Hospital of Strasbourg, Nouvel Hôpital Civil, 1 place de l’Hôpital, BP 426-67091, Strasbourg 67000, France; Research Unit - UR3074, Translational Cardiovascular Medicine, University of Strasbourg, Strasbourg, France; Groupe pour l’Enseignement et la Recherche Cardiovasculaire en Alsace (GERCA), Strasbourg, France; Hanoï Medical University, Hanoï, Vietnam

**This editorial refers to “Aspirin does not modify cardiovascular event risk in endometriosis in the California Teachers Study”, by A. Seitz *et al*., https://doi.org/10.1093/ehjopen/oeaf023.**

Endometriosis is a chronic neuroinflammatory disorder defined by the presence of endometrial-like tissue and lesions outside the uterus. Affecting an estimated 190 million people worldwide,^1^ this debilitating disorder impacts around 10% of reproductive-age women and is notably prevalent among those experiencing infertility. Endometriosis is commonly linked to markedly impaired quality of life and can present with a broad clinical spectrum, ranging from asymptomatic to severely debilitating forms. Diagnosis is typically delayed, and existing therapies—such as surgical removal of lesions, which often repeat, and pharmacological interventions, which can be burdened by adverse effects—offer only limited relief. Significant attention in recent years has focused on the association between endometriosis and an increased risk of atherosclerotic cardiovascular disease (ASCVD).^2^ This relationship raises important questions regarding the mechanistic and molecular links between the two conditions, while providing physicians with limited guidance on mitigating this risk in clinical practice.

In this issue of European Heart Journal Open, Seitz *et al.*^3^ tackle the burning issue of aspirin for primary prevention of ASCVD in endometriosis. The authors provide valuable insights by confirming an elevated risk of major adverse cardiovascular events (MACE), particularly among younger women (<40 years old) with self-reported endometriosis. These findings, derived from the California Teachers Study (CTS) with a cohort of over 120 000 female teachers, underscore the need for early cardiovascular monitoring in endometriosis, well before the onset of menopause. Furthermore, they highlight that endometriosis should be viewed not merely as a niche gynaecological or reproductive issue, but as a significant public health concern with far-reaching cardiovascular and systemic implications.

Beyond describing heightened risk, Seitz *et al.* question whether commonplace therapies like aspirin and statins might modify cardiovascular outcomes among individuals with endometriosis. The results, though sobering, are instructive: neither medication class appeared to protect against the elevated risk. The mention of statin use lacks sufficient detail, as neither the intensity of statin therapy nor the corresponding LDL-C levels were reported. Furthermore, the optimal target and threshold for LDL-C reduction in the context of primary prevention among patients with endometriosis have yet to be clearly established. Intriguingly, women who used aspirin, regardless of endometriosis status, demonstrated a higher incidence of MACE. This underscores the possibility of confounding by indication or other unmeasured cardiovascular risks in those with aspirin use. Seitz *et al.* challenge the prevailing cardiological assumption that antiplatelet therapy—long studied for primary prevention in other conditions—may also benefit patients with endometriosis. These negative findings regarding aspirin, while disappointing, are nonetheless somewhat reassuring from a mechanistic standpoint. They highlight the inherent complexity of endometriosis pathophysiology, characterized by numerous, intertwined molecular pathway, and underscore the likelihood that standard cardiovascular prophylaxis may not fully address the distinct risk mechanisms involved.

The association between endometriosis and cardiovascular disease raises important mechanistic questions. Chronic low-grade inflammation, dysregulated immune function, and hormonal disturbances are hallmarks of endometriosis. These factors possibly interacting with genetic variants may predispose to endothelial dysfunction and accelerate atherosclerosis. Psychological stress and chronic pain might also exacerbate cardiometabolic vulnerability. Given that the role of hormonal therapies, non-steroidal anti-inflammatory drugs (NSAIDs), and surgical interventions in modulating cardiovascular outcomes is likewise unclear, more research is clearly warranted.

Although the CTS offer a robust dataset, it is subject to inherent limitations. Because endometriosis and medication use were self-reported at periodic intervals, the data are susceptible to recall bias and lack key details such as disease severity and specific medication dosages or duration. Furthermore, the CTS time frame (1995–2020) may have disproportionately captured severely symptomatic endometriosis cases, predating the current era of heightened awareness; fuelled by mass media, patient advocacy, and broader clinical recognition; along with advances in diagnostics, such as magnetic resonance imaging and salivary microRNA signatures. Patient heterogeneity remains an inherent limitation of the CTS design, given that ∼85% of participants were non-Hispanic white women, and detailed information on endometriosis grading, severity, history, and treatment was not available. Non-steroidal anti-inflammatory drugs are considered a first-line therapy for endometriosis-associated pain. However, given their well-established adverse cardiovascular effects, the potential interaction between NSAID use and the potential cardioprotective benefits of aspirin could not be addressed in the current analysis. Robust investigation will be key to elucidating the cardiovascular risks linked to NSAID therapy in endometriosis.

Yet, these limitations do not overshadow the central message: the association between endometriosis and cardiovascular disease is both real and clinically significant. Cardiologists should regard endometriosis as more than merely a gynaecological concern. Screening for cardiometabolic risk factors in younger patients, particularly those with severe endometriosis, may be prudent. Moreover, integrating gynaecological, cardiovascular, and primary care expertise could be pivotal in preventing downstream cardiovascular complications.

Looking ahead, we need robust, longitudinal research including randomized controlled trials to determine exactly which treatments, if any, can reduce cardiovascular risk in endometriosis. Anti-inflammatory therapies, hormonal modulators, interventional procedures, and lifestyle interventions may prove beneficial, but each must be rigorously evaluated in the context of this complex disease (*Figure 1*). Endometriosis is not confined to the pelvis; it carries systemic implications that clinicians and researchers can no longer overlook. At the policy level, it may be time for major international cardiology societies to jointly establish a dedicated ‘cardio-endometriosis council’: a collaborative nucleus focused on investigating a condition affecting an estimated 10% of reproductive-age women and linked to adverse cardiovascular outcomes. This is an opportune time to embrace integrated and collaborative strategies that address this under recognized risk, ensuring individuals with endometriosis have consistent access to comprehensive cardiovascular care.

**Figure 1:**
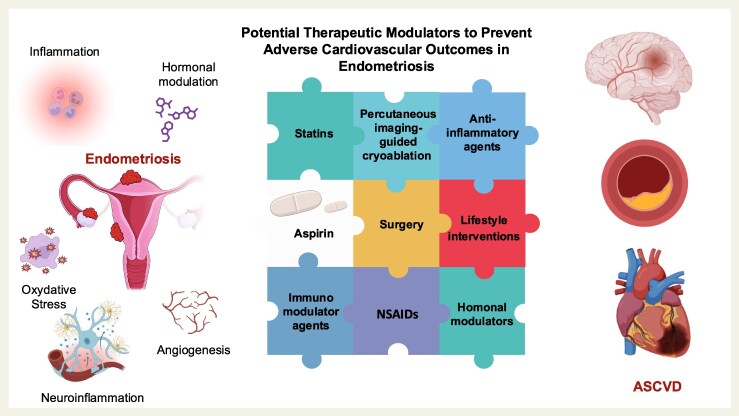
Potential therapeutic modulators to prevent adverse cardiovascular outcomes in endometriosis. Endometriosis is a chronic gynaecological disorder driven by complex molecular interactions that underlie its pathogenesis. Several strategies and therapeutic modulators may help mitigate the heightened risk of atherosclerotic cardiovascular disease in affected women. However, further investigation is required to establish the most effective therapeutic approach, which will likely involve synergistic modulation through multiple agents. Created with BioRender.com. ASCVD, atherosclerotic cardiovascular disease; NSAIDs, non-steroidal anti-inflammatory drugs.

## References

[1] World Health Organization. *Endometriosis*. Geneva: World Health Organization; 2023. Retrieved from https://www.who.int/news-room/fact-sheets/detail/endometriosis

[2] Havers-Borgersen E, Hartwell D, Ekelund C, Butt JH, Østergaard L, Holgersson C, Schou M, Køber L, Fosbøl EL. Endometriosis and long-term cardiovascular risk: a nationwide Danish study. Eur Heart J 2024;45:4734–4743. 39219447 10.1093/eurheartj/ehae563

[3] Seitz A, Zhang C, Bull L, Kamel H, White H, Navi BB, Shin JH, Berkin J, Kaiser JH, Liao V, Liberman AL. Aspirin does not modify cardiovascular event risk in endometriosis in the California. Eur Heart J Open 2025. Available from: 10.1093/ehjopen/oeaf023. PMC12076410 40370503

